# The differential effects of tumor burdens on predicting the net benefits of ssCART-19 cell treatment on r/r B-ALL patients

**DOI:** 10.1038/s41598-021-04296-3

**Published:** 2022-01-10

**Authors:** Minghao Li, Sheng-Li Xue, Xiaowen Tang, Jiayu Xu, Suning Chen, Yue Han, Huiying Qiu, Miao Miao, Nan Xu, Jingwen Tan, Liqing Kang, Zhou Yu, Xiaoyan Lou, Yang Xu, Jia Chen, Zhiqiang Yan, Weixing Feng, Depei Wu, Lei Yu

**Affiliations:** 1 Institute of Biomedical Engineering and Technology, Shanghai Engineering Research Center of Molecular Therapeutics and New Drug Development, School of Chemistry and Molecular Engineering, East China Normal University, NO, 3663 North Zhongshan Road, Shanghai, 200065 China; 2 National Clinical Research Center for Hematologic Diseases, Jiangsu Institute of Hematology, The First Affiliated Hospital of Soochow University, Suzhou, China; 3 Institute of Blood and Marrow Transplantation, Collaborative Innovation Center of Hematology, Soochow University, Suzhou, China; 4 Shanghai Unicar-Therapy Bio-Medicine Technology Co., Ltd, No 1525 Minqiang Road, Shanghai, 201612 China; 5 Institute of Intelligent System and Bioinformatics, College of Intelligent Systems Science and Engineering, Harbin Engineering University, Harbin, 150001 Heilongjiang China

**Keywords:** Acute lymphocytic leukaemia, Adaptive immunity, Immunotherapy

## Abstract

The tumor burden (TB) is significantly related to the severity of cytokine release syndrome (CRS) caused by CAR-T cells, but its correlation with therapeutic efficacy has not been systematically studied. This study focused on the effects of the TB level on both the safety and efficacy of ssCART-19 as a treatment for r/r B-ALL. Taking the 5% tumor burden as the boundary, the study participants were divided into 2 groups, high and low tumor burden groups. Under this grouping strategy, the impacts of differential r/r B-ALL TBs on the clinical therapeutic efficacy (CR rate and long-term survival) and safety profiles after ssCART-19 cell treatment were analysed. 78 patients were reported in this study. The differential B-ALL TBs significantly affected the complete remission (CR) rates of patients treated with ssCART-19, with rates of 93.94% and 75.56% in the low and high TB groups, respectively (*P* = 0.0358). The effects of TBs on long-term therapeutic efficacy were further studied based on event-free survival (EFS) and overall survival (OS) profiles; both the OS and EFS of the low TB group were better than those of the high TB group, but the differences were not statistically significant. Importantly, the time points of TB measurement did not significantly affect the OS and EFS profiles regardless of whether the TBs were measured before or after fludarabine-cyclophosphamide (FC) preconditional chemotherapy. On the other hand, the severity of CRS was significantly correlated with the TB level (*P* = 0.0080), and the incidence of sCRS was significantly related to the TB level (the sCRS incidence increased as the TB level increased, *P* = 0.0224). Unexpectedly, the ssCART-19 cell expansion peaks were not significantly different (*P* = 0.2951) between the study groups. Patients with a low r/r B-ALL TB yield more net benefits from CAR-T treatment than those with a high TB in terms of safety and CR rate. These findings are critical and valuable for determining the optimal CAR-T cell treatment window for r/r B-ALL patients and will further the development of comprehensive and reasonable CAR-T cell treatment plans for r/r B-ALL patients with differential TBs.

Trial registration: ClinicalTrials.gov identifier, NCT03919240.

## Introduction

As a new immunotherapy, chimeric antigen receptor T (CAR-T) cell therapy has shown remarkable effects in refractory or relapsed B-cell acute lymphoblastic leukemia (r/r B-ALL). Studies have shown that the complete remission (CR)/CR with incomplete haematologic recovery (CRi) rate of CAR-T cell therapy can be as high as 90%^1,2^. However, despite the continuous development of CAR-T cell therapy, some insufficiencies remain regarding its long-term effectiveness and safety. For example, recurrence can occur in patients (more than 50%) treated with CAR-T cells within 12 months after the achievement of CR^3^. Moreover, serious side effects, such as cytokine release syndrome (CRS) and neurotoxicity, often occur during CAR-T cell therapy and can even cause patient death^4–8^. These problems have largely limited the clinical application of CAR-T cell therapy in r/r B-ALL. Therefore, identifying predictive and early indicators of the clinical efficacy, prognosis, and safety of CAR-T cells is of great significance for formulating a reasonable clinical CAR-T cell treatment plan.

Multiple studies have shown that numerous factors are potentially related to the efficacy of CAR-T cells^9–14^. For instance, Pan J reported that the threshold dose for CAR-T cell infusion was related to cell expansion and therapeutic efficacy^15^, and higher remission rates (55–94.3%) were shown to be achieved with doses exceeding 1 × 10^6^/kg^3^. In addition, Fraietta, J. A found higher expression levels of memory-related genes in CAR-T cells from responsive chronic lymphocytic leukemia (CLL) patients, while the genes with increased expression in CAR-T cells from nonresponsive patients were shown to be involved in effector cell differentiation, glycolysis, depletion, and apoptosis^15^. Qin, J. S also found that ibrutinib could transform CAR-T cells into memory cells, which thereby increased the clearance rate of CD19+ tumors in mice and prolonged the survival times of tumor-bearing mice^16^. Although some studies have reported that tumor burden factors are correlated with CR after CAR-T cell therapy and with the survival rate, their inconsistent patient treatment methods and CAR-T cell infusion doses potentially affect the accuracy of statistical analyses^17^. Therefore, standardizing and systematically studying the tumor burden impacts on the CAR-T cell treatment response and long-term efficacy are necessary and important.

Severe CRS (sCRS) and CAR-T cell-related neurotoxicity are common side effects of CAR-T cell treatment. Many factors have been reported to be associated with these side effects, including the dose used for CAR-T cell reinfusion, the costimulatory domain molecules on CAR-T cells, and the types and amounts of proinflammatory factors released^8,11,12,17–19^. Studies have also shown that the tumor burden is significantly related to the safety of CAR-T cell clinical treatment^6,20^. Davila, M. L analysed the correlations among 39 cytokines, the tumor burden prior to treatment and sCRS and found that the increased levels of 7 cytokines (interferon (IFN)-γ, interleukin (IL)-5, IL-6, IL-10, FIt-3L, granulocyte macrophage-colony stimulating factor (GM-CSF) and fractalkine) were related to the pretreatment tumor burden and to sCRS. Further analysis showed that the severity of CRS was significantly correlated with the tumor burden prior to 19-28z CAR-T cell infusion^19^. Similarly, Brentjens, R. J reported that the cytokine levels were correlated with the B-ALL tumor burden before treatment^21^. Moreover, Teachey, D. T found high levels of 24 cytokines, including IFNγ, IL6, sgp130, and sIL6R, within the first month after CAR-T cell infusion, which implied an intricate and high correlation with CRS^7^.

Based on the duality of the efficacy and side effects of CAR-T cell treatment, the ultimate benefit of CAR-T cell treatment depends on the net benefit yielded from balancing treatment efficacy and side effects. The tumor burden affects the safety of CAR-T cell clinical treatment, but its impact on CAR-T cell efficacy has not been investigated in a systematic and standardized manner. Therefore, we analysed the therapeutic outcomes of patients with r/r B-ALL treated with ssCART-19 cells, researching the correlations of the tumor burden with the CR rate, long-term survival (overall survival (OS) and event-free survival (EFS)), and clinical risk. This study aimed to elucidate reliable indicators that are predictive of clinical treatment efficacy and risks and to guide the formulation of a reasonable overall clinical treatment plan for patients with varying tumor burdens to maximize the benefit of CAR-T cell treatment.

## Patient and methods

### Study design and patient enrolment

This study was approved by the ethics committee of the First Affiliated Hospital of Soochow University and conducted to assess the safety and efficacy of ssCART-19 cells (i.e., T cells expressing a CAR composed of an anti-CD19 single-chain antibody fragment with IL-6-specific shRNA) in patients with relapsed or refractory CD19+ B-cell malignancies. The primary endpoint was the safety of treating relapsed or refractory CD19+ B-cell malignancy patients with ssCART-19 cells, and adverse events were graded according to the Common Terminology Criteria for Adverse Events (CTCAE) version 4.0.3. The secondary endpoint was the objective response rate at the end of the study. All the patients provided written informed consent, and the enrolled patients met the following inclusion criteria: diagnosed with CD19+ r/r B-ALL with sufficient organ function (left ventricular ejection fraction ≥ 0.5 as determined by echocardiography, creatinine < 1.6 mg/dL, aspartate aminotransferase/aspartate aminotransferase < 3 × the upper limit of normal, bilirubin < 2.0 mg/dL) and a Karnofsky performance status ≥ 60 or an ECOG performance status ≤ 2. The study was registered at ClinicalTrials.gov (NCT03919240). First Submitted Date: 15/04/2019. First Posted Date: 18/04/2019.

All patients underwent bone marrow evaluations before or after interim therapy and immediately before the T cell infusion.

### Protocol treatment and assessments

After leukapheresis and ssCART-19 cell manufacturing, patients received conditioning chemotherapy consisting of fludarabine (30 mg per square metre of body surface area per day) and cyclophosphamide (Cy; 500 mg per square metre per day) on days − 5, − 4, and − 3 (FC treatment) before the administration of ssCART-19 cells at a dose of 5 × 10^6^ cells per kilogram of body weight with split doses of 10%, 30%, and 60%. The patient's body temperature, blood pressure, heart rate and pulse oxygen were monitored, and blood was drawn according to the clinical trial process to monitor the patient's cytokine and CAR-T cell expansion levels in the peripheral blood. Blood test parameters, blood biochemistry parameters, ferritin, and C-reactive protein were also routinely monitored according to the protocol.

### Response and toxicity assessment

CR was defined as less than 5% blasts in the bone marrow without myelosuppression, no circulating blasts in peripheral blood, and the absence of extramedullary disease, regardless of cell count recovery. A negative MRD status was defined as less than 0.01% bone marrow blasts assessed by multiparameter flow cytometry, and absence of genetic aberrants assessed by karyotype analysis or molecular detection. Relapsed disease was defined as loss of CR status after the achievement of CR, including hematological relapse (bone marrow ALL blasts > 5%) or extramedullary relapse (central nervous system, other site)^17^. The analysis of overall survival used death as the event, and the analysis of event-free survival used the earliest of no response, relapse, or death as the event. Patients who did not have an event had their data censored for the analyses at the date at which they were last known to be alive.

CRS was graded according to the National Cancer Institute CTCAE version 4.03 and was considered to be severe at grades higher than or equal to 3.

CAR-T cell-related neurotoxicity was assessed according to the National Cancer Institute CTCAE version 4.03, and severe neurotoxic effects were defined as a seizure of any grade or a toxic effect of grade 3 or higher.

### Manufacture of ssCART-19 cell products

The ssCART-19 cell manufacturing procedures were described previously^22^. Briefly, peripheral blood mononuclear cells were purified from the patient’s blood by gradient centrifugation using Lymphoprep (Oriental Hua Hui, Beijing, China), and their CD3+ T cells were then enriched by positive selection using magnetic bead separation (Miltenyi Biotec, Bergisch Gladbach, Germany). The T cells were transduced with lentiviral supernatant, followed by activation in vitro using anti-CD3/CD28 monoclonal antibodies (Miltenyi Biotec) at 5% CO2 and 37 °C for 24 h. After transduction for 48 h, the CAR-T cells were cultured and expanded in AIM-V T cell medium (Gibco, Grand Island, NY, USA), which contained 100 IU/ml recombinant human IL-2 (PeproTech, Rocky Hill, NJ, USA), 5 ng/ml recombinant human IL-7 (PeproTech), 5 ng/ml recombinant human IL-15 (PeproTech) and 10% autologous plasma, at 5% CO2 and 37 °C for 14 days.

### Cytokine concentration assessment

Cytokines were measured using the Th1/Th2 Cytometric Bead Array Kit II (BD Bioscience) according to the manufacturer’s instructions. In brief, serum was collected from patients at different time points. The cytokine-capture microspheres were first mixed and then incubated with serum samples and fluorophore-labelled antibodies for 3 h before being washed and evaluated by flow cytometry (Thermo Fisher). The concentration of each cytokine was calculated from standard curves.

### Quantification of ssCART-19 cell expansion and maintenance

The expansion and persistence of the ssCART-19 cells were monitored by real-time quantitative PCR (qPCR). All patients peripheral blood samples have obtained at different time points. The corresponding DNA has amplified with primers and probes complementary to specific sequences within the lentiviral vector followed by extracted from the peripheral blood samples. qPCR has performed using the ABI 7500 Real-Time PCR System (Applied Biosystems, Thermo Fisher Scientific, USA), and calculation of the CAR-T copy number depends on the standard curve established with the plasmid encoding the transgene^23^.

### Flow cytometry assays

CAR-T cell differentiation stages were assessed by flow cytometry. CAR-T cells were harvested and washed twice with 1 ml of phosphate-buffered saline containing 2% foetal bovine serum (FBS; Gibco), followed by incubation with the following antibodies: APC-Cy7-conjugated anti-CD8 (344714, BioLegend, California, USA), Alexa Fluor 700-conjugated anti-CD4 (56004942, eBioscience), PE-conjugated anti-CCR7 (353204, BioLegend), PerCP-Cy5.5-conjugated anti-CD45RA (304122, BioLegend), and PE-Cy7-conjugated anti-CD127 (25-1287-42, eBioscience)^24^.

### Statistical analyses

The EFS and OS data in this study were analysed using the Kaplan–Meier log-rank test. T tests (paired or unpaired), the chi-square test and Fisher's exact test were also used in this study. P values less than 0.05 indicated statistical significance.

### Ethics approval and consent to participate

This study was performed in accordance with the Declaration of Helsinki and was approved by the ethics review committee of the First Affiliated Hospital of Soochow University. All participants provided written informed consent.

## Results

### Patient characteristics

**Figure 1:**
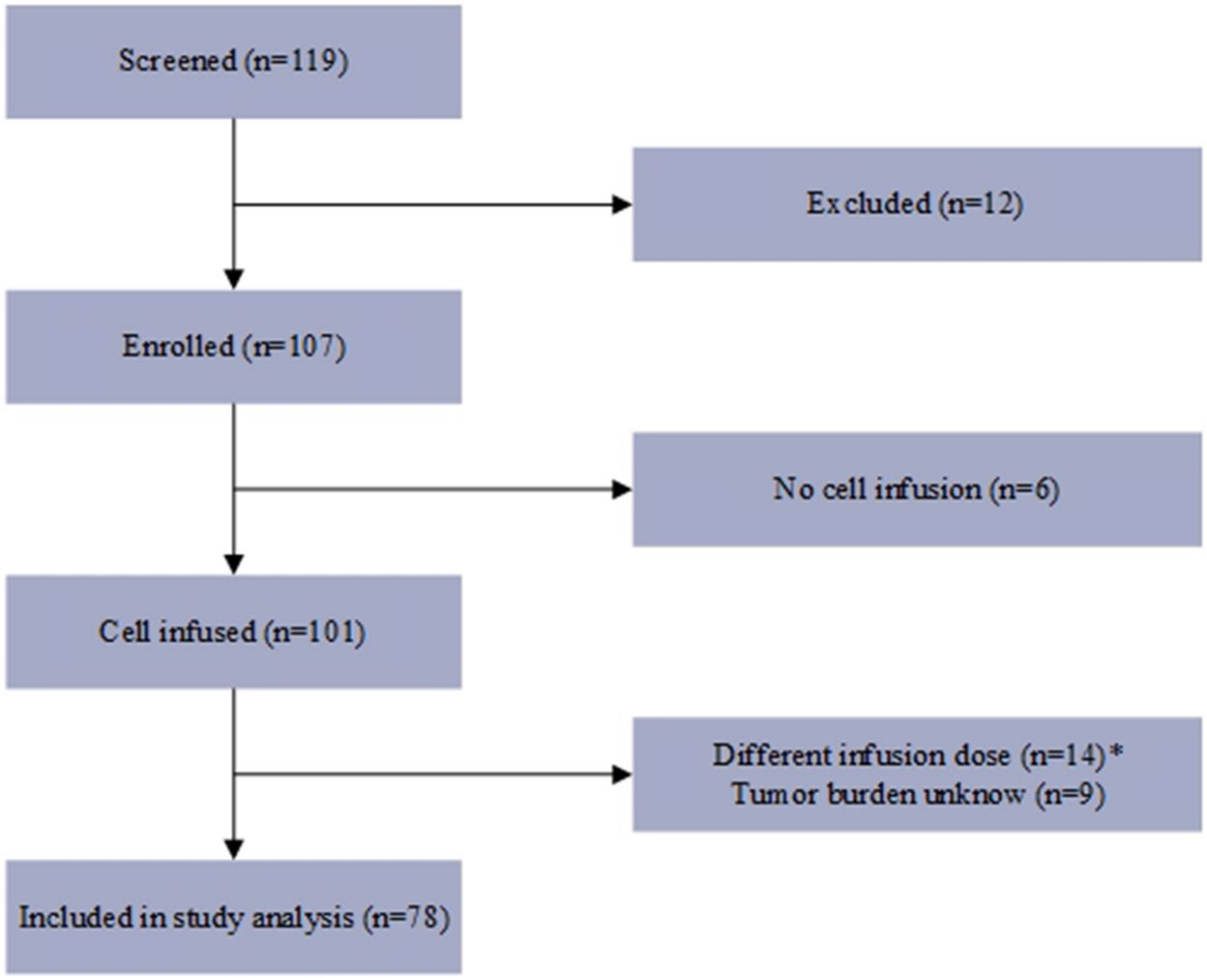
Flow chart of the study participants. The diagram shows the courses all of the study participants from the time of consent to treatment. *In this study, only patients with an infusion dose of 5 × 10^6^ cells/kg were included in the statistical analysis.

A total of 107 patients with r/r B-ALL were enrolled between February 2017 and May 2020. Among them, 78 patients were treated with ssCART-19 cells and were included in the statistical analysis, while the remaining 29 patients were not included in the analysis. The specific exclusion criteria are shown in Fig. 1.

All 78 patients were treated with FC. According to the patient's tumor burden before CAR-T cell reinfusion, the 78 patients were divided into two groups. Thirty-three patients were included in the low tumor burden group and had minimal residual disease (MRD) with bone marrow blast percentages ranging from 0.01 to less than 5; the remaining 45 patients were in the high tumor burden group, with bone marrow blasts percentages equalling or exceeding 5.

Among the 33 patients in the low tumor burden group, 25 (75.76%) received ssCART-19 cell therapy as a third or later salvage treatment, 26 (78.79%) were treated with other approaches for more than half a year, and 11 (33.33%) received bone marrow transplantation before ssCART-19 cell treatment. Nine (23.27%) patients were diagnosed with Philadelphia chromosome (Ph)-positive ALL, 28 (84.85%) relapsed before ssCART-19 cell treatment, 1 (3.03%) had a TP53 gene mutation, and 11 (33.33%) were evaluated as poor cytogenetic risk patients.

Among the 45 patients in the high tumor burden group, 30 (66.67%) received ssCART-19 cell therapy as a third or later salvage treatment, 28 (62.22%) were treated with other approaches for more than half a year, and 8 (17.78%) received bone marrow transplantation before ssCART-19 cell treatment. Five (11.11%) patients were diagnosed with Ph-positive ALL, 29 (64.44%) relapsed before ssCART-19 cell treatment, 2 (4.44%) had a TP53 gene mutation, and 13 (28.89%) were evaluated as poor cytogenetic risk patients. Table 1 includes additional baseline patient information.

**Table 1:** Patient and treatment characteristics.

Characteristic		Tumour burden*		*P* value
		Low	High	
Patient No		33	45	NA
Age	Median (Range)	32 (10,73)	30 (9,68)	0.4283
Sex	Male, n (%)	15 (45.45)	23 (51.11)	0.6531
Medical history	Ph+, n (%)	9 (23.27)	5 (11.11)	0.0799
	≥ 6 months, n (%)	26 (78.79)	28 (62.22)	0.1415
	Pre-chemotherapy ≥ 3 times, n (%)	25 (75.76)	30 (66.67)	0.4563
	Pre-HSCT, n (%)	11 (33.33)	8 (17.78)	0.1811
	Relapse, n (%)	28 (84.85)	29 (64.44)	0.0695
Tumour burden evaluation time	Before FC, n (%)	25 (75.76)	32 (71.11)	0.7972
	After FC, n (%)	8 (24.24)	13 (28.89)	
TP53 mutation	Yes, n (%)	1 (3.03)	2 (4.44)	> 0.9999
Cytogenetic risk	Poor, n (%)	11 (33.33)	13 (28.89)	0.8046

Ph+ , Philadelphia chromosome-positive; Pre-HSCT, previous haematopoietic stem cell transplantation; TP53, tumor protein P53; FC, fludarabine and cyclophosphamide.

*A high tumor burden was defined as a bone marrow blast level of 5% or more, and a low disease burden was defined as a bone marrow blast level of less than 5%. Minimal residual disease was assessed by multiparameter flow cytometry^25^, and a negative minimal residual disease status was defined as a bone marrow blast level of less than 0.01%.

It is worth noting that the time of tumor burden evaluation in this study was not completely uniform. The evaluation time points were distributed before and after FC treatment. Among the study cohort, the tumor burdens of 25 (75.76%) people in the low tumor burden group were evaluated prior to FC treatment, while those of 32 (71.11%) people in the high tumor burden group were evaluated before FC treatment. According to the results of this study, the time point of tumor burden assessment had no significant effect on the long-term efficacy (Fig. 3).

### Response rates and long-term survival

**Figure 2:**
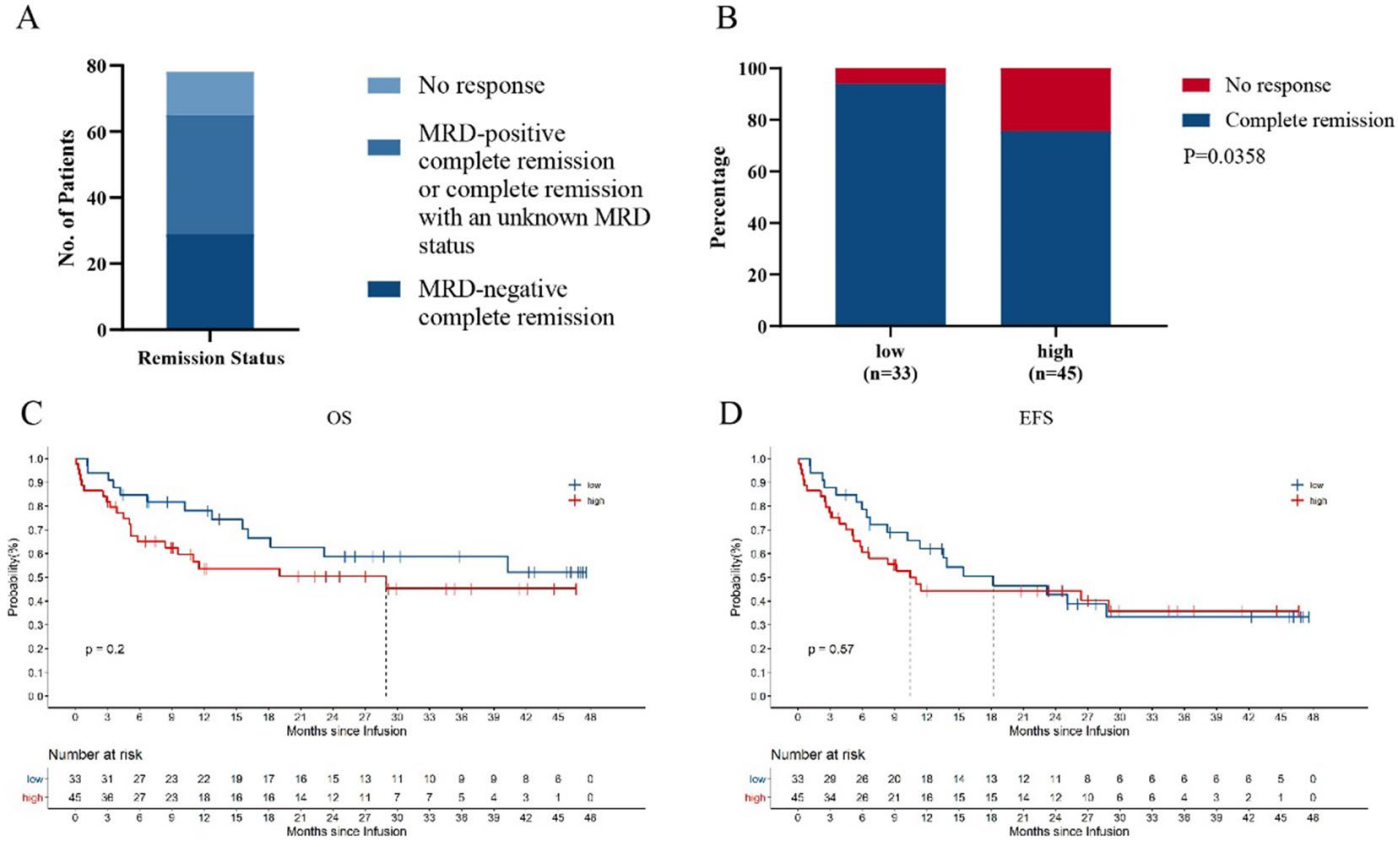
Response to ssCART-19 cells. (**A**) The rates of minimal residual disease (MRD)-positive complete remission, MRD-negative complete remission and no response. Patients with an unknown MRD status were included with those who achieved MRD-positive complete remission. (**B**) Correlation between the remission status and tumor burden (*P* = 0.0358; Fisher's exact test). (**C, D**) Kaplan–Meier analyses demonstrated better OS and EFS rates in patients who had a low tumor burden (blue line, n = 33) than in those who had a high tumor burden (red line, n = 45; *P* = 0.2 and *P* = 0.57, respectively; log-rank test).

Among 78 patients, 29 achieved MRD-negative CR (37.18%), 36 achieved MRD-positive or MRD status-unknown CR (46.15%), and 13 patients had no response (NR) (16.67%). The overall CR rate reached 83.33% (Fig. 2A).

Analysis of the high and low tumor burden groups revealed that the CR rate was significantly correlated with the tumor burden. The CR rate of the low tumor burden group was significantly higher than that of the high tumor burden group (*P* = 0.0358, Fig. 2B). Analysis of the long-term survival data revealed that the long-term efficacy was also related to the tumor burden. Although the difference was not significant, the OS of patients in the low tumor burden group was better than of patients in the high tumor burden group, and the EFS within 21 months of patients in the low tumor burden group was better than that of patients in the high tumor burden (Fig. 2C,D).

**Figure 3:**
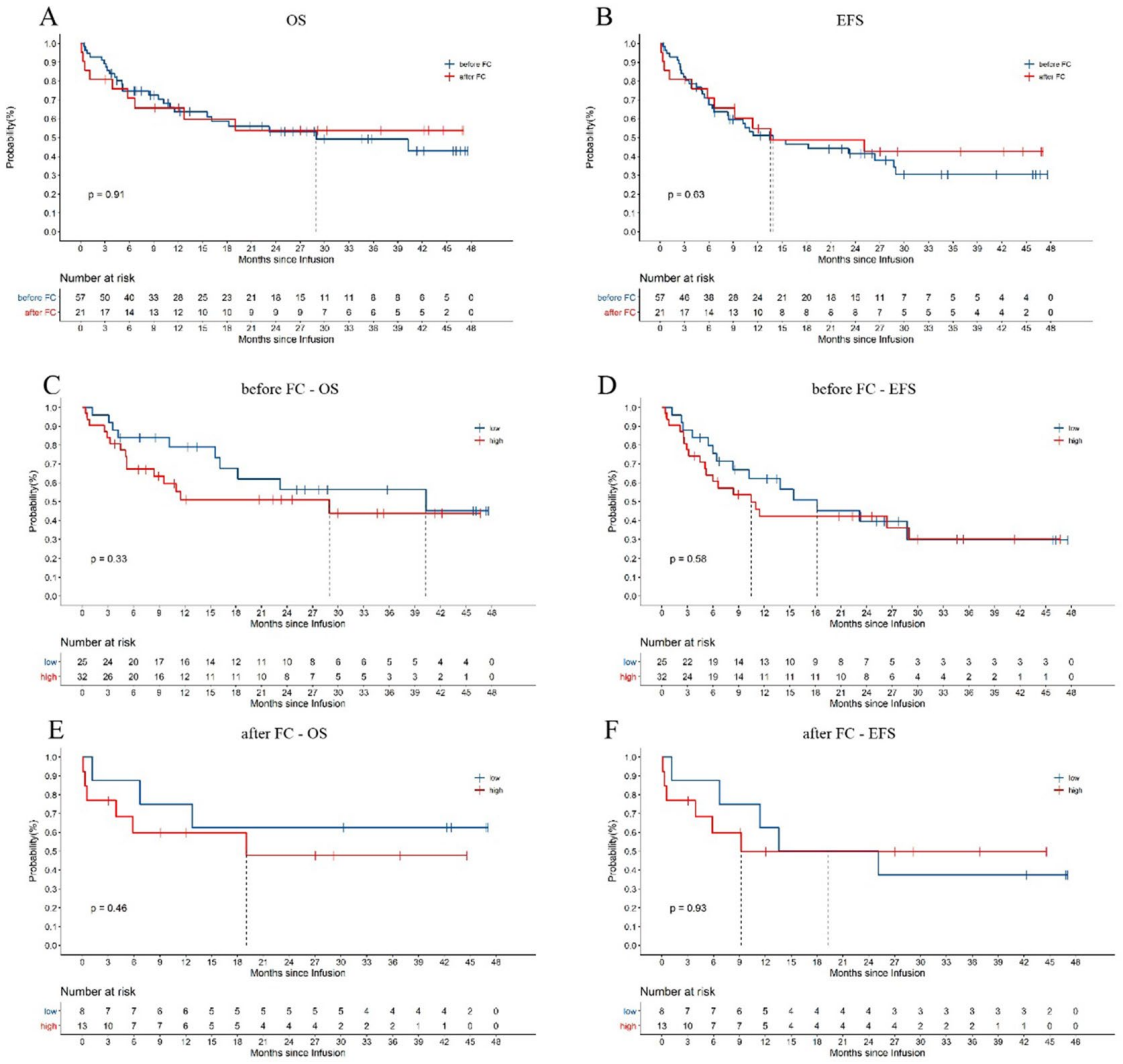
Long-term survival based on the tumor burden evaluation time point. (**A**, **B**) The OS and EFS, respectively, of patients who underwent bone marrow evaluations before and after interim therapy. (**C**, **D**) Correlations between OS or EFS and the tumor burden evaluated before FC treatment. (**E**, **F**) Correlations between OS or EFS and the tumor burden evaluated after FC treatment.

The time points of bone biopsy to assess the patients’ tumor burdens were not completely uniform due to the clinical specificities. Therefore, patient data were obtained from tumor burdens measured both before and after FC treatment in the study. To explore whether this difference affected the statistical results, we conducted grouping statistical analysis according to whether the tumor burden was assessed before or after FC treatment. According to our statistical results, the time point of tumor burden assessment did not affect the statistical results regarding OS and EFS after ssCART-19 cell treatment (Fig. 3A,B). Additionally, the tumor burden groups were further evaluated under this stratification, and the OS and EFS trends were consistent with those shown in Fig. 2C,D for the tumor burdens obtained both before and after FC treatment (Fig. 3C,F). These results suggest the tumor burden measurements both before and after FC treatment can be used as a reference in the clinic.

### Safety analysis

**Figure 4:**
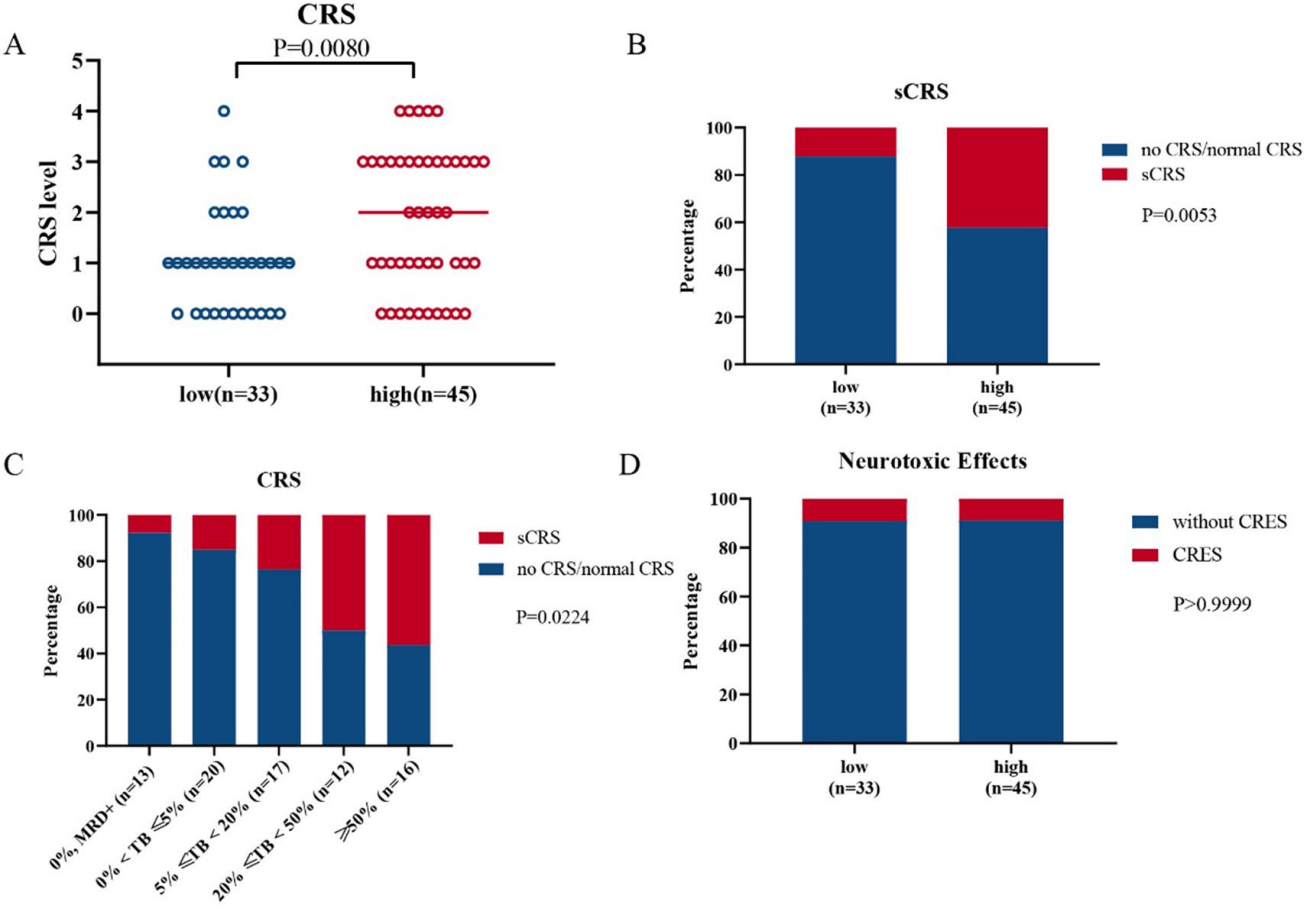
Cytokine release syndrome and neurotoxic effects after infusion of ssCART-19 cells. (**A**) Correlation between the CRS level and tumor burden (*P* = 0.0080; t test). (**B**) Correlation between the occurrence of sCRS and the tumor burden (*P* = 0.0053; Fisher's exact test). (**C**) Correlation between the occurrence of sCRS and the tumor burden subgroup (*P* = 0.0224; one-way ANOVA); no CRS means CRS level 0, normal CRS means CRS levels 1 and 2, and sCRS means CRS level 3 or higher. (**D**) No correlation was observed between the occurrence of neurotoxic effects and the tumor burden (P > 0.9999; Fisher's exact test).

Statistical analysis showed a significant positive correlation between the tumor burden and the level of CRS response during treatment, which was basically consistent with the conclusion reported by Davila ML^19^. After mapping the CRS response level and the tumor burden groupings, the high tumor burden group had an increased CRS response and a higher CRS response level, thereby also indicating a higher incidence of sCRS, than the low tumor burden group (Fig. 4A,B). According to the specific tumor burden assessments, the patients were further divided into 5 subgroups based on their tumor burden measurements: (a) 0% tumor burden with MRD positivity; (b) 0% < tumor burden < 5%; (c) 5% ≤ tumor burden < 20%; (d) 20% ≤ tumor burden < 50%; and e) tumor burden ≥ 50%. The increasing tumor burden gradient in the tumor burden subgroups reflected an obvious correlation with sCRS and could therefore provide a reference for the clinical prevention of sCRS (Fig. 4C). There was no difference in neurotoxicity among the groups. It is worth emphasizing that ssCART-19 cells are suitable for the treatment of central nervous system leukemia. This lack of a difference among groups might be related to the intrinsic characteristics of ssCART-19 cells, which could reduce neurotoxicity and improve safety (Fig. 4D).

### Cytokine release

We conducted a more in-depth analysis of patients’ blood samples to further explore the causes of the phenomenon resulting from the clinical treatment described above.

**Figure 5:**
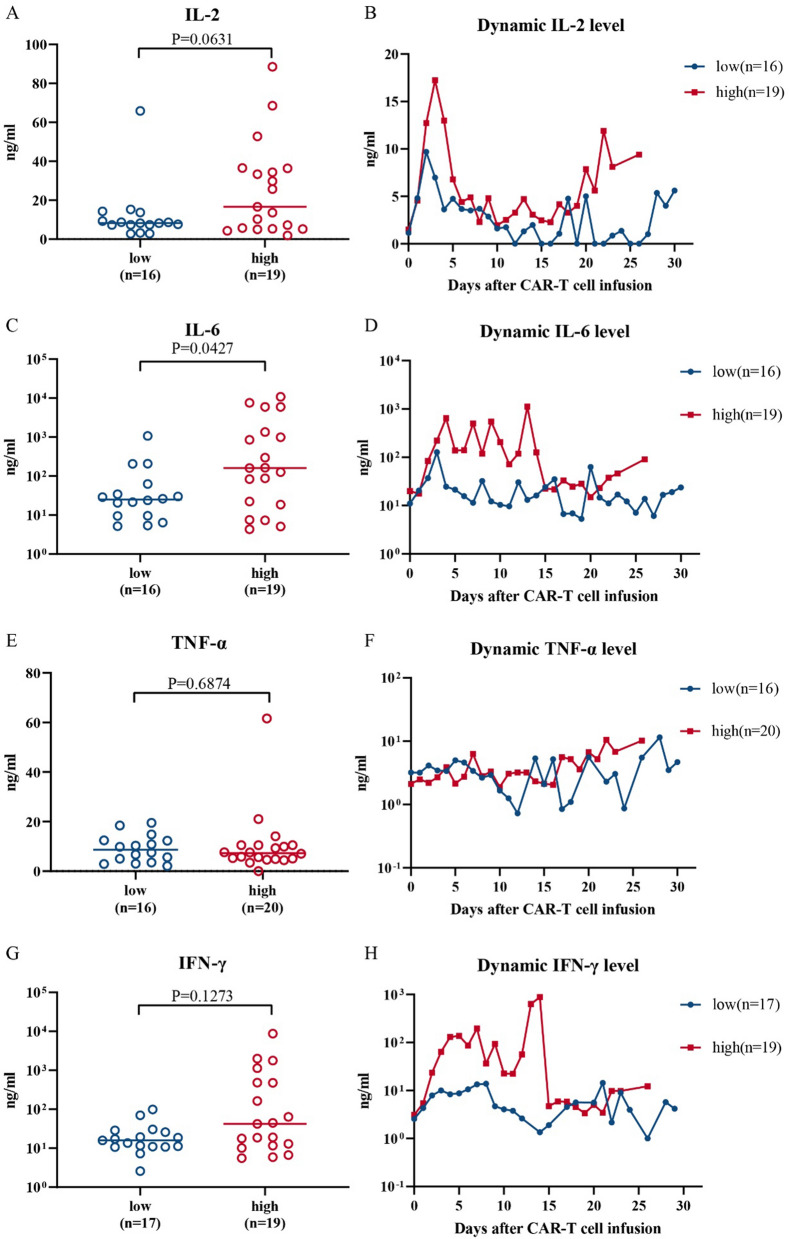
Cytokines and the tumor burden. (**A**, **C**, **E**, **G**) The peak concentration scatter plots of IL-2, IL-6, TNF-α and IFN-γ. (**B**, **D**, **F**, **H**) Plots of the dynamic levels of IL-2, IL-6, TNF-α and IFN-γ.

We studied the CRS response according to the protocol established by Marco L. Davila^4^ by investigating four cytokines that are potentially related to CRS: IL-2, IL-6, TNF-α and IFN-γ. A higher level of only the cytokine IL-6 was significantly correlated with the tumor burden (Fig. 5A,C,E,G). The amount of IL-6 released in the high tumor burden group was significantly higher than that released in the low tumor burden group (Fig. 5C, *P* = 0.0427). IL-6 is closely related to the CRS response^22^, suggesting that it is the main factor underlying the high sCRS response rate in the high tumor burden group. Based on the dynamic results for these four cytokines, like TNF-α, IL-2, IL-6 and IFN-γ were secreted at higher levels in the high tumor burden group than in the low tumor burden group, but the differences were not obvious (Fig. 5B,D,F,H). This result suggests that patients in the high tumor burden group had a higher safety risk during the treatment process than those in the low tumor burden group.

### Correlation between the tumor burden and post-infusion CAR-T cell expansion

**Figure 6:**
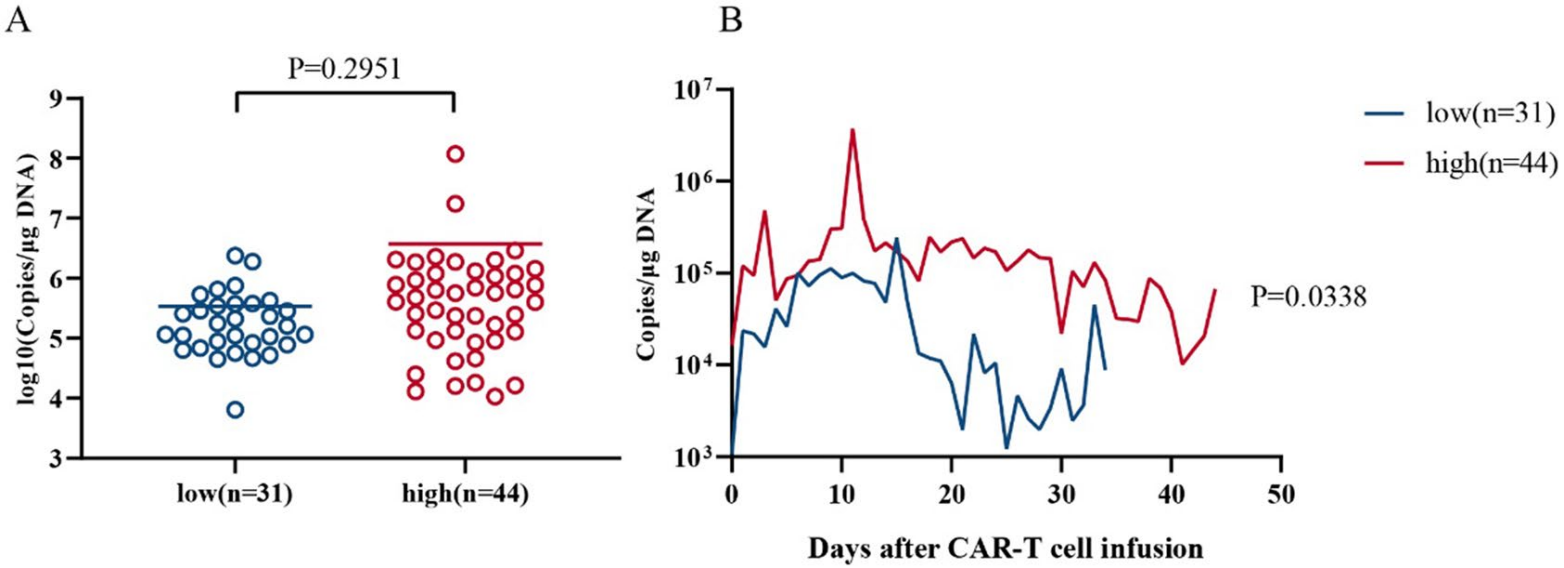
Post-infusion CAR-T cell expansion and the tumor burden. (**A**) Correlation between chimeric antigen receptor T (CAR-T) cell expansion after infusion and the tumor burden (*P* = 0.2951). (**B**) CAR-T cell counts in the blood over the first 44 days after CAR-T cell infusion in patients with a low tumor burden (blue line) compared with patients with a high tumor burden (red line).

No significant correlation between the peak CAR-T cell expansion and the tumor burden was observed, suggesting that ssCART-19 cells are mild and stable in vivo. Although the average peak copy number of the high tumor burden group was higher than that of the low tumor burden group (Fig. 6A), the CAR-T cells did not overreact due to the large number of target antigens in the high tumor burden group, which improved the safety of the treatment. This result might also explain why the CR rate was significantly lower in the high tumor burden group than in the low tumor burden group.

CAR-T cell dynamics were plotted on a curve and analysed using a paired t test. Although the copy numbers were assessed at different times, the overall statistical analyses showed significant differences (Fig. 6B). This analysis showed that patients with different tumor burdens responded differentially to ssCART-19 cell therapy, but the responses were not overly intense, thereby ensuring effective treatment while taking safety into account.

## Discussion

CAR-T cell therapy triggers a cellular immune response via the specific activation of CAR-T cells by specific antigens on target cells. Therefore, the number of target cells affects the degree of CAR-T cell stimulation and the intensity of the immune response. B-ALL is a type of haematological tumor, and the tumor burden is equivalent to the target cell load. Therefore, the B-ALL tumor burden is one of the main factors that determines the strength of the bodily CAR-T cell-target cell-mediated cellular immune response and the performance and clinical efficacy of CAR-T cells during clinical treatment. The CAR-T cell treatment of B-ALL in patients with a high tumor burden is more likely to cause sCRS than that of patients with a low tumor burden^6,19–21^. sCRS is a systemic inflammatory response to CAR-T cell treatment caused by the release of cytokines from infused CAR-T cells and can lead to widespread reversible organ dysfunction^26^. Clear conclusions have not been drawn from the current research on whether the tumor burden affects the efficacy of CAR-T cells. Park, J. H concluded that patients with a high disease burden had a shorter long-term survival than those with a low disease burden^17^, while Brentjens, R. J reported that the tumor burden was not correlated with clinical efficacy^21^. These differential results may be related to the specific treatment plans and number of clinical cases. For the study of Park, J. H, the different treatment processes for patients which might have a significant impact on the clinical data. Firstly, 53 patients enrolled in the study, among them 43 patients received cyclophosphamide (Cy) conditioning chemotherapy before CAR-T infusion, and another 10 patients received Fludarabine + Cy chemotherapy. Secondly, the CAR-T infusion dosage was also different, 33 patients received different dosages of CAR-T cells. Some patients with a higher tumor burden received a lower dose of 1 × 106 19-28z CAR-T cells/kg. Some patients with a lower tumor burden received a higher dose of 3 × 106 19-28z CAR-T cells/kg. For the study of Brentjens, R. J, only 5 patients were treated during the study, from a statistical point of view, it is hard to obtain an accurate conclusion with this limited number of study subjects. Because CRS often occurs only during CAR-T cell treatment, the correlation between the tumor burden and CRS is easily studied. In contrast, drawing effective conclusions from the correlations of the tumor burden with EFS and OS often requires a sufficient number of patients and a long follow-up time. Therefore, a more systematic and reasonable approach to analysing clinical research data is critical and necessary.

In this study, 78 patients were treated with ssCART-19 cells and we systematically evaluated baseline parameters in the study participants in a standardized manner prior to the statistical analysis to ensure that the baseline data were consistent and that the various tumor burden analyses were as reliable as possible. At the same time, we followed a consistent ssCART-19 treatment process including the conditioning chemotherapy, CAR-T dosage to reduce the impacts of factors other than the tumor burden.

Based on the above research and our research results, we conclude that ssCART-19 cells have good curative effects and that the CR rate is significantly related to the tumor burden. The OS and EFS results demonstrated that patients with a low tumor burden benefited more than those with a high tumor burden. Park, J. H^17^ reportedly observed no significant correlation between the tumor burden and the CR rate, but the tumor burden did significantly affect the OS and EFS. Although the conclusions of the two studies are not completely consistent, the overall efficacy (both CR rate and OS/EFS) trends are the same, and despite the significant differences, both studies suggest that a lower tumor burden leads to higher CR rate and better OS/EFS. Furthermore, this conclusion is basically consistent with our previous study^27^. The differential conclusions may be due to the different treatment plans utilized in the two studies. The pretreatment chemotherapy regimens were different for the patients in the high and low tumor burden groups, and the CAR-T cell doses were also different. These differences can obviously significantly impact the treatment effects^15^. Brentjens, R. J reported that the tumor burden was not related to clinical efficacy^21^. We believe that his study did not include a sufficient number of patients, thereby leading to a lack of sufficient data to draw definitive conclusions.

The impact of the tumor burden on the safety of the treatment process observed herein is consistent with previous reports^6,19–21^. We also found that the incidence of sCRS in patients with a high tumor burden was significantly higher than that in patients with a low tumor burden, indicating a lower safety risk of CAR-T cell therapy in patients with a low tumor burden. Additionally, we found a significant correlation between the IL-6 peak and tumor burden, which potentially explains why patients with a high tumor burden are more likely to develop sCRS. Based on this, we also further divided the patients into 5 subgroups according to their tumor burden, and the CRS reaction became increasingly severe as the tumor burden increased. This finding has not yet been reported and may provide a more accurate clinical reference which may serve as an important early warning and estimated reference value for the occurrence of sCRS risk during clinical CAR-T cell treatment.. In addition, we statistically analysed the number of adverse events during the clinical treatment process, and the results were positively correlated (Fig. S2), suggesting that the relationship between adverse events and the tumor burden is basically consistent with that between CRS and the tumor burden. Laboratory indicators such as red blood cells, hemoglobin, lymphocyte percentage, C-reactive protein, ferritin, albumin, and γ-glutamyl transpeptidase levels are also correlated with the tumor burden (Fig. S3). Similarly, Pan, J found that patients had fever and elevated ALT/AST levels and that the incidence of hypoxemia and coagulopathy were potentially related to the leukemia burden^14^.

We also herein investigated the pharmacokinetics of CAR-T cells in r/r B-ALL patients with varying tumor burdens, and the CAR-T cell expansion peak was not significantly correlated with the tumor burden. Therefore, ssCART-19 cells are mild and stable in vivo. Although the number of CAR-T cells in the high tumor burden group was higher than that in the low tumor burden group, the cells did not overreact even with the high tumor burden and more target antigens, demonstrating the improved safety of ssCART-19 cells. This result might also explain why the CR rate in the high tumor burden group was significantly lower than that in the low tumor burden group. A dynamic CAR-T cell curve was analysed using a paired t test. Although the copy numbers were determined at different time points, the overall statistical results showed significant differences. This result demonstrates that while ssCART-19 cells prompt differential responses in patients with different tumor burdens, the response are not overly intense, thereby ensuring effective treatment while considering safety. Unlike other reports^10,28,29^, this study did not identify a correlation between CAR-T cell pharmacokinetics and the severity of CRS (Fig. S4). These results suggest that the treatment of patients with a high tumor burden is not as safe as that of patients with a low tumor burden, which may not simply be related to the number of CAR-T cells.

This study revealed that the tumor burden is a significantly important reference for the efficacy and safety of CAR-T cell treatment. Before CAR-T cell infusion, the tumor burden should be reduced to the greatest extent possible to improve the treatment effect and reduce the safety risk. In addition to conventional chemotherapy, chemotherapy combined with radiotherapy can potentially be utilized to improve the treatment effect, thereby reducing the tumor burden. For example, we have attempted to use chemotherapy and radiotherapy to reduce the high burden in patients with diffuse large B-cell lymphoma (DLBCL) before CAR-T cell treatment (clinical trial number NCT03196830), and found that radiotherapy is highly recommended before CAR-T cell treatment for R/R DLBCL patients^30^.

For extremely refractory patients, other methods to reduce the tumor burden may not be effective, and clinicians can consider adjusting the treatment plan to improve the treatment effect and reduce the safety risk. For example, Memorial Sloan-Kettering Cancer Center (MSKCC) and Fred Hutchinson Cancer Research Center (FHCRC) adopt risk-adapted CAR-T cell dosing strategies to choose the best dose for balancing efficacy and safety^1,12,19,21^. Most of these centres use a “split dose” regimen to reduce the clinical risk, and patients may benefit more when this strategy is utilized.

The impacts of other factors on survival were evaluated when assessing the patients’ baseline information in this study, but no significant correlations were found (data not shown). Whether the tumor burden assessments at different time points, such as before and after FC pretreatment, have differential effects on the CAR-T cell treatment response, severe CRS, EFS, and OS was previously unknown. However, this study showed that under uniform FC pretreatment conditions, assessing the tumor burdens before and after FC treatment did not affect the final statistical results (Fig. 3; Fig. S1). This provides flexibility for clinicians to assess the benefits of CAR-T cell clinical treatment when tumor burden detection at different time points is required.

Finally, the sample size may not be large enough after more subgroup are divided, therefore, the more case data are needed to consolidate our research conclusions. And with the more related factors and more types of cytokines related to the CRS reported, the cytokines and biochemistry marker reported in this study may not be enough. Further studies may require more testing items.

## Conclusion

The CAR-T cell treatment of r/r B-ALL patients with a low tumor burden has a obviously higher CR rate than that of patients with a high tumor burden. The time point of tumor burden assessment does not affect the predictions of CR rate, survival time and safety (incidence of sCRS) based on the tumor burden. When making an overall CAR-T cell treatment plan, minimizing the tumor burden may become an important goal. The results of this study provide an important basis for formulating a reasonable plan for the CAR-T cell treatment of r/r B-ALL to obtain the greatest benefits. They may also provide an important basis for rationally formulating an overall CAR-T cell treatment plan for other cancers to obtain greater benefits.

## Supplementary Information

Supplementary Information.

## Data Availability

The data-sets used and/or analysed during the current study are available from the corresponding author on reasonable request.
