# Supplementary material for: The differential effects of tumor burdens on predicting the net benefits of ssCART-19 cell treatment on r/r B-ALL patients
Source: Sci Rep. 2022 Jan 10;12:378. doi: 10.1038/s41598-021-04296-3 (PMC8748521; doi:10.1038/s41598-021-04296-3)
Supplement: Supplementary file 1 — Supplementary Information. [file 41598_2021_4296_MOESM1_ESM.pdf]

Supplementary information

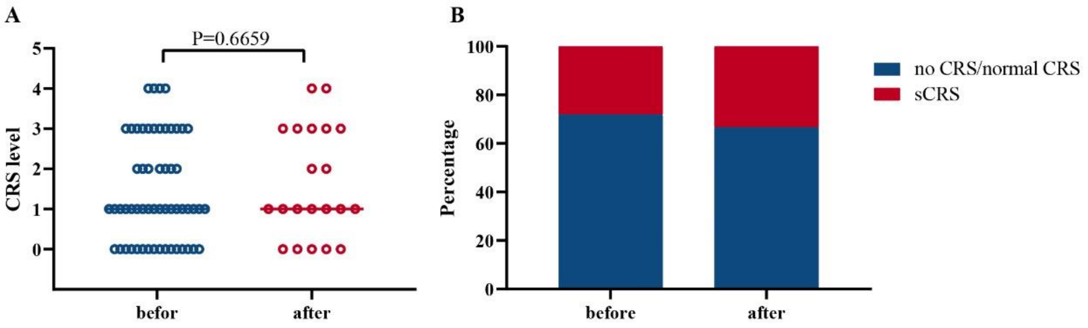

Additional file 1: Figure S1. Correlation between the CRS and tumor burden evaluation time point. **(A)** Correlation between the CRS level and tumor burden evaluation time point (blue indicates before FC; red indicates after FC). **(B)** Correlation between the sCRS occurrence rate and tumor burden evaluation time point. No CRS means CRS level 0, normal CRS means CRS level 1 and 2, sCRS means CRS level 3 or higher.

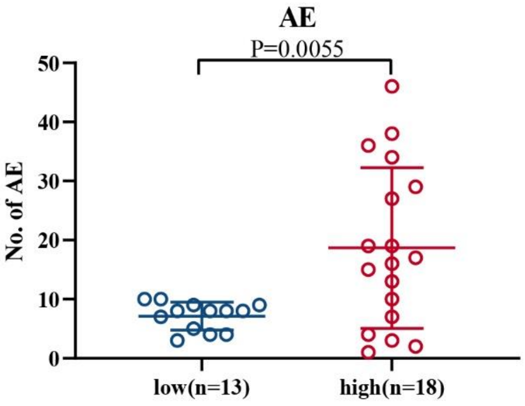

Additional file 2: Figure S2. Correlation between the AE counts and tumor burden (P=0.0055; t test).

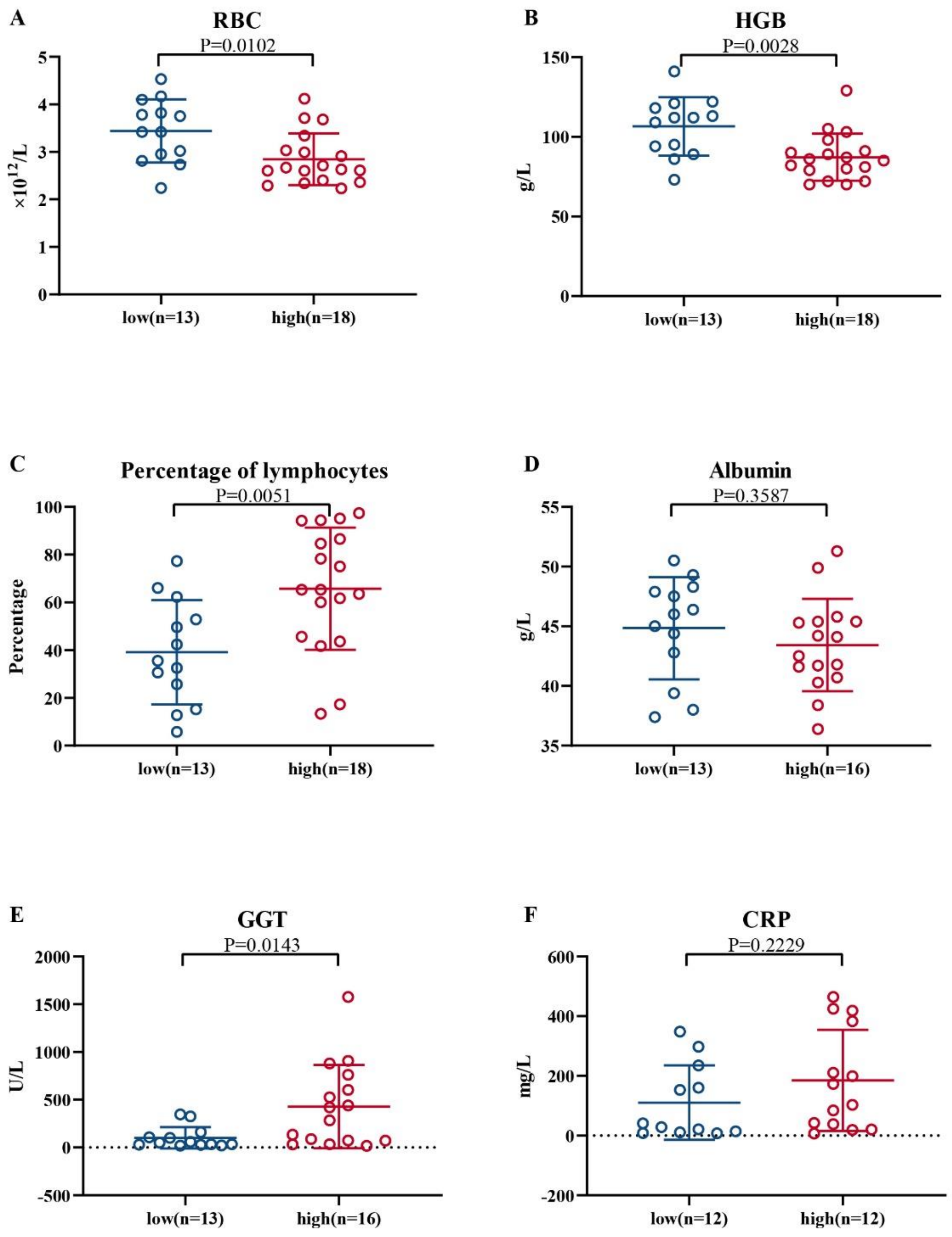

Additional file 3: Figure S3. Correlation between the laboratory tests and tumor burden. **(A)** Red blood cells (RBCs), **(B)** hemoglobin (HGB), **(C)** percentage of lymphocytes, **(D)** albumin, **(E)** γ-glutamyl transpeptidase (GGT), and **(F)** C-reactive protein (CRP). The RBCs, HGB, percentage of lymphocytes and GGT showed significant correlations with the tumor burden.

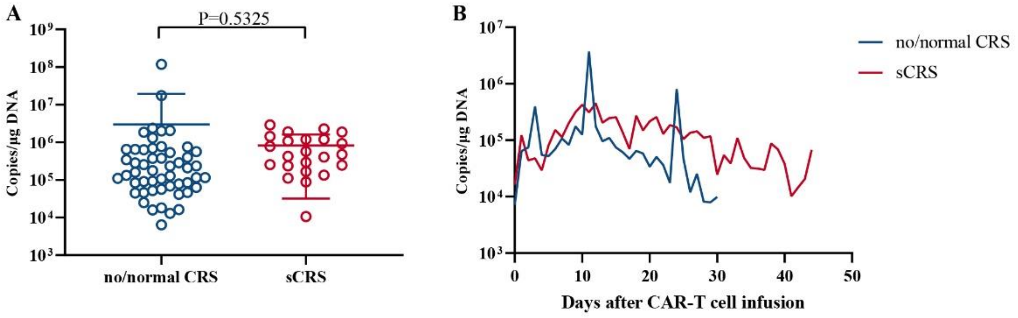

Additional file 4: Figure S4. Post-infusion CAR-T Cell expansion and CRS. **(A)** Correlation between chimeric antigen receptor (CAR) T-cell expansion after infusion and the CRS status (P=0.5325). **(B)** CAR T-cell counts in the blood over the first 44 days after CAR-T cell infusion for patients with no/normal CRS (blue line) and patients with sCRS (red line).
